# The clinical features, underlying immunology, and treatment of autoantibody‐mediated movement disorders

**DOI:** 10.1002/mds.27446

**Published:** 2018-09-14

**Authors:** Valentina Damato, Bettina Balint, Anne‐Kathrin Kienzler, Sarosh R. Irani

**Affiliations:** ^1^ Oxford Autoimmune Neurology Group, Nuffield Department of Clinical Neurosciences University of Oxford Oxford UK; ^2^ Institute of Neurology, Department of Neuroscience Catholic University Rome Italy; ^3^ Department of Neurology University Hospital Heidelberg Germany; ^4^ Oxford University Hospitals John Radcliffe Hospital Oxford UK

**Keywords:** Neuroimmunology, autoimmune encephalitis, autoantibody, stiff person syndrome, immunology

## Abstract

An increasing number of movement disorders are associated with autoantibodies. Many of these autoantibodies target the extracellular domain of neuronal surface proteins and associate with highly specific phenotypes, suggesting they have pathogenic potential. Below, we describe the phenotypes associated with some of these commoner autoantibody‐mediated movement disorders, and outline increasingly well‐established mechanisms of autoantibody pathogenicity which include antigen downregulation and complement fixation. Despite these advances, and the increasingly robust evidence for improved clinical outcomes with early escalation of immunotherapies, the underlying cellular immunology of these conditions has received little attention. Therefore, here, we outline the likely roles of T cells and B cells in the generation of autoantibodies, and reflect on how these may guide both current immunotherapy regimes and our future understanding of precision medicine in the field. In addition, we summarise potential mechanisms by which these peripherally‐driven immune responses may reach the central nervous system. We integrate this with the immunologically‐relevant clinical observations of preceding infections, tumours and human leucocyte antigen‐associations to provide an overview of the therapeutically‐relevant underlying adaptive immunology in the autoantibody‐mediated movement disorders. © 2018 The Authors. Movement Disorders published by Wiley Periodicals, Inc. on behalf of International Parkinson and Movement Disorder Society.

The spectrum of autoantibody‐mediated movement disorders includes a broad and clinically heterogeneous group of conditions. The movement disorders occur either in isolation or, more commonly, as prominent and often distinctive manifestations of autoimmune encephalitides. Patients typically present with a subacute onset and multifocal neurological features involving the cortex, basal ganglia, brain stem, and/or spinal cord (Table 1). Although formal epidemiological data are still emerging, it is clear that both sexes and patients of all ages can be affected by this spectrum of disorders. The detection of neuronal autoantibodies in serum and the CSF can help to guide the diagnostic process, prognosis, and the treatment of these disorders. In addition, the autoantibody specificity may predict an underlying tumour association (Table 1). Perhaps most important, many of these conditions respond to immunotherapies, making them one of the earliest therapeutic considerations in the correct clinical context.^1^, ^2^, ^3^, ^4^, ^5^

**Table 1:** Antibody associations with movement disorders and tumors

Antigen	Movement disorders	Additional features	Tumour association
Extracellular antigens			
NMDA receptor	Orobuccolingual dyskinesia, catatonia, limb dystonia, stereotypies, chorea	Amnesia, psychiatric features, seizures, dysautonomia, coma	Ovarian teratoma (especially if > 18 years old)
LGI1	Faciobrachial dystonic seizures, myoclonus, chorea, parkinsonism	LE, hyponatremia	Thymoma, SCLC
CASPR2	Chorea, ataxia	LE, Morvan's syndrome, neuromyotonia, neuropathic pain	Thymoma
GABA_B_ receptor	Ataxia, OMS, chorea	LE	SCLC
GABA_A_ receptor	OMS, SPS, chorea	Status epilepticus, LE	Thymoma, SCLC
mGluR1	Ataxia	Seizures, cognitive impairment	Hodgkin Lymphoma, renal cancer
VGCC	Ataxia	Lambert‐Eaton syndrome	SCLC
DPPX	PERM, OMS, tremor, ataxia	Behaviour changes, cognitive decline, seizures, dysautonomia, diarrhoea, weight loss	B cell neoplasms
IgLON5	Chorea, parkinsonism, ataxia, limb stiffness, dystonia	Non‐REM and REM‐sleep disorder, stridor, bulbar symptoms, cognitive impairment, eye movement abnormalities	Not reported
Glycine receptor	SPSD	Seizures, encephalopathy	Thymoma, lymphoma, SCLC, breast cancer
Dopamine 2 receptor	Chorea, dystonia, parkinsonism, tics	Psychiatric disturbances	Not reported
Neurexin‐3α	Orofacial dyskinesias	Confusion, seizures, decrease level of consciousness	Not reported
Intracellular antigens			
Amphiphysin	SPSD		SCLC, breast cancer
GAD65	SPSD, ataxia	LE, epilepsy	Rare: thymoma, lymphoma, breast cancer, other
CRMP5	Chorea, ataxia, OMS	LE, encephalomyelitis, neuropathies	SCLC, thymoma
Ma2	OMS, parkinsonism	LE, brain stem encephalopathy	Testicular cancer
Ri	Jaw dystonia, ataxia, OMS, parkinsonism	Brain stem encephalopathy	SCLC, breast cancer
Yo	Ataxia		Ovarian cancer, breast cancer
Hu	Ataxia	LE, polyneuropathy, brainstem encephalopathy, pseudoathetosis	SCLC
Tr/DNER	Ataxia		Hodgkin Lymphoma
GFAP	Tremor, ataxia	Encephalopathy, meningitis, myelopathy, seizures, dysautonomia, psychiatric	Ovarian teratoma, prostate adenocarcinoma

NMDA, *N*‐methyl‐d‐aspartate; LGI1, leucine‐rich glioma‐inactivated 1; CASPR2, contactin‐associated protein‐like 2; GABA_A/B_, gamma‐aminobutyric acid A/B; mGluR1, metabotropic glutamate receptor type 1; VGCC, voltage gated calcium channel; DPPX, dipeptidyl‐peptidase‐like protein‐6; GAD, glutamic acid decarboxylase; CRMP5, collapsin‐response mediated protein 5; GFAP, glial fibrillary acidic protein; SCLC, small cell lung cancer; LE, limbic encephalitis; SPS, stiff person syndrome; SPSD, stiff‐person syndrome spectrum disorder; PERM, progressive encephalomyelitis with rigidity and myoclonus; OMS, opsoclonus myoclonus syndrome; REM, rapid eye movement.

The practical importance of both the antigenic specificity and the effects of immunotherapies make our understanding of the underlying immunopathology critical to managing patients with these conditions.^4^, ^6^, ^7^, ^8^, ^9^, ^10^ Therefore, in this review, we focus on the immunological mechanisms that are likely to initiate and propagate the diseases and outline roles for the autoantibody‐producing plasma cells, their precursor B cells, and Tcells. We also discuss the relevance of antigen‐drainage, tumors, the blood‐brain barrier and theprinciple pathogenic mechanisms by which the autoantibodies may induce disease at a molecular level(Figs. 1 and 2).^1^, ^4^, ^6^ This permits us to consider methods to tailor immunotherapies toward the underlying immunology. However, to ensure the accurate administration of immunotherapies, we describe these alongside the key clinical features that permit early recognition of immunotherapy‐responsive autoantibody‐mediated movement disorders.^3^, ^5^, ^7^ Throughout, we focus on the most common likely pathogenic autoantibodies and review those autoantibodies which potentially challenge the paradigm that targetting of surface epitopes implies causation.

**Figure 1:**
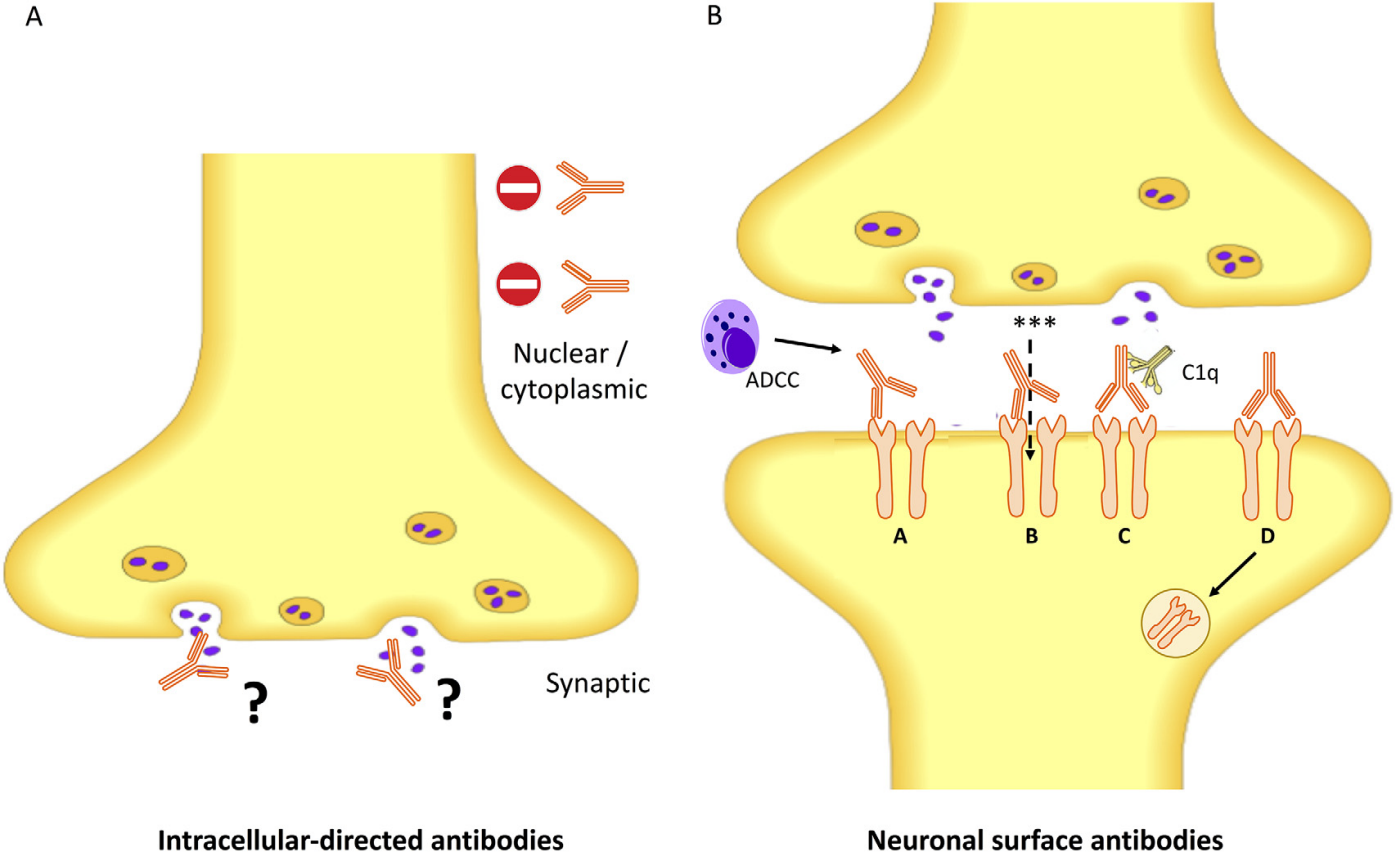
Autoantibodies directed at intracellular and extracellular domains of neuronal proteins. (A) Autoantibodies against constitutive nuclear or cytoplasmic proteins do not appear to gain access to their targets, whereas those directed against predominantly intracellular synaptic proteins may gain access at the time of vesicle fusion. (B) Pathogenic mechanisms of neuronal surface proteins. Neuronal surface proteins have a direct pathogenic effect on the antigen through various mechanisms: (A) antibody‐dependent cell‐mediated cytotoxicity (ADCC), (B) direct target modulation through agonist/antagonist effects, (C) complement activation, and (D) antigen internalization. * = ions; red circle with horizontal white line denotes a “no entry” sign; C1q, complement component 1q.

**Figure 2:**
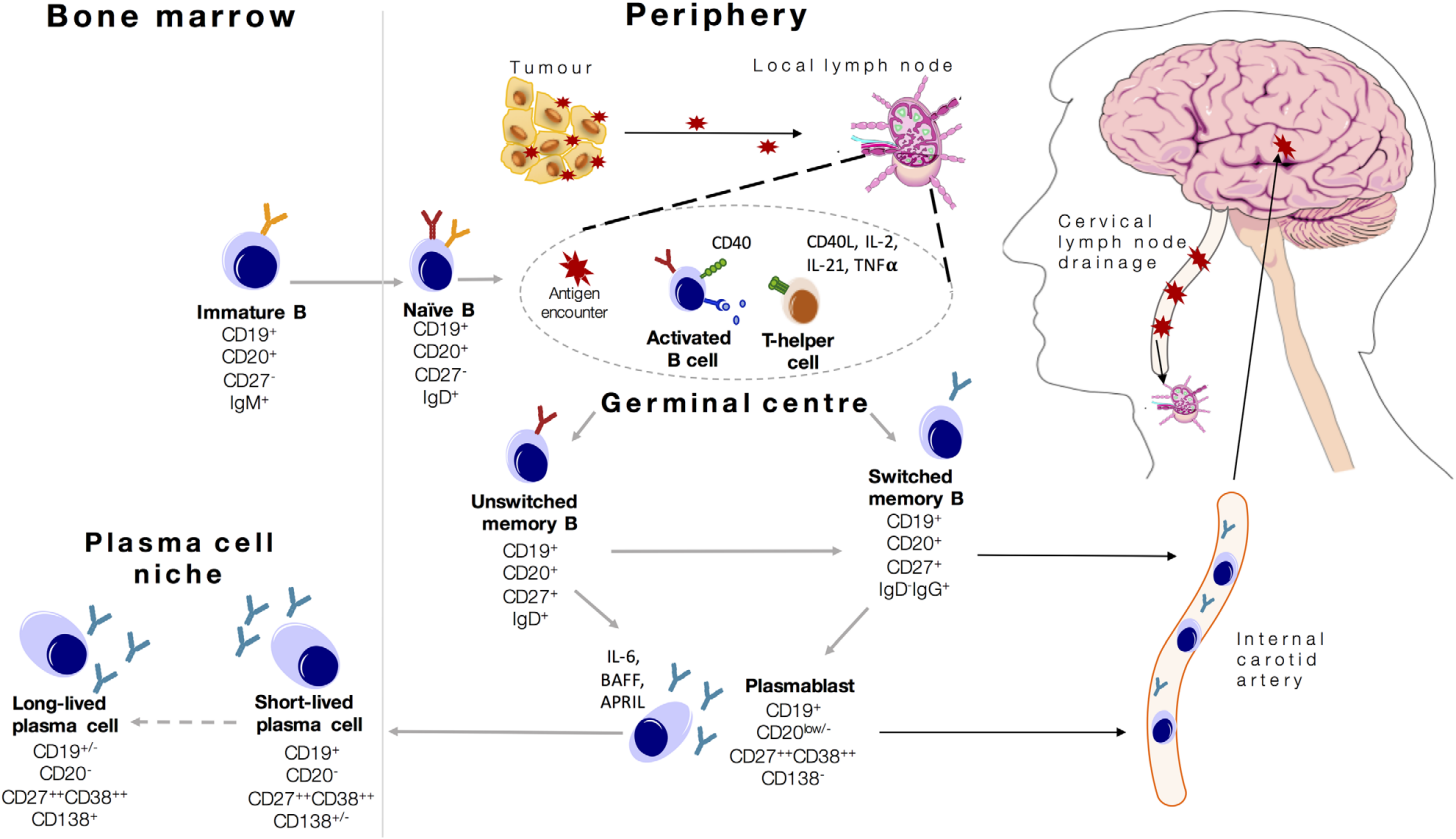
Model of B cell activation in neuronal surface autoantibody‐associated movement disorders. Triggers of immunological activation in CNS autoimmunity may lead to exposure of antigen (red star) and its presentation in the germinal centres of the cervical lymph nodes. Interaction between naïve B cells and CD4^+^ T helper cells in germinal centres causes maturation of B cells into antigen‐specific cells that can switch their immunoglobulin chain to express IgG. These cells can subsequently differentiate into antibody‐secreting plasmablasts and become tissue‐resident plasma cells. Circulating memory B cells and plasmablasts can reach the CNS through the internal carotid artery, re‐encounter the antigen, and produce antibodies (intrathecal synthesis). Modified with permissions from Wilson et al.^83^ IgG (immunoglobulin G), IL‐2 (interleukin 2), IL‐21 (interleukin 21), IgD (Immunoglobulin D), TNF alpha. CD = cluster of differentiation.

## Inside or Outside the Plasma Membrane? Location Matters

**Figure 3:**
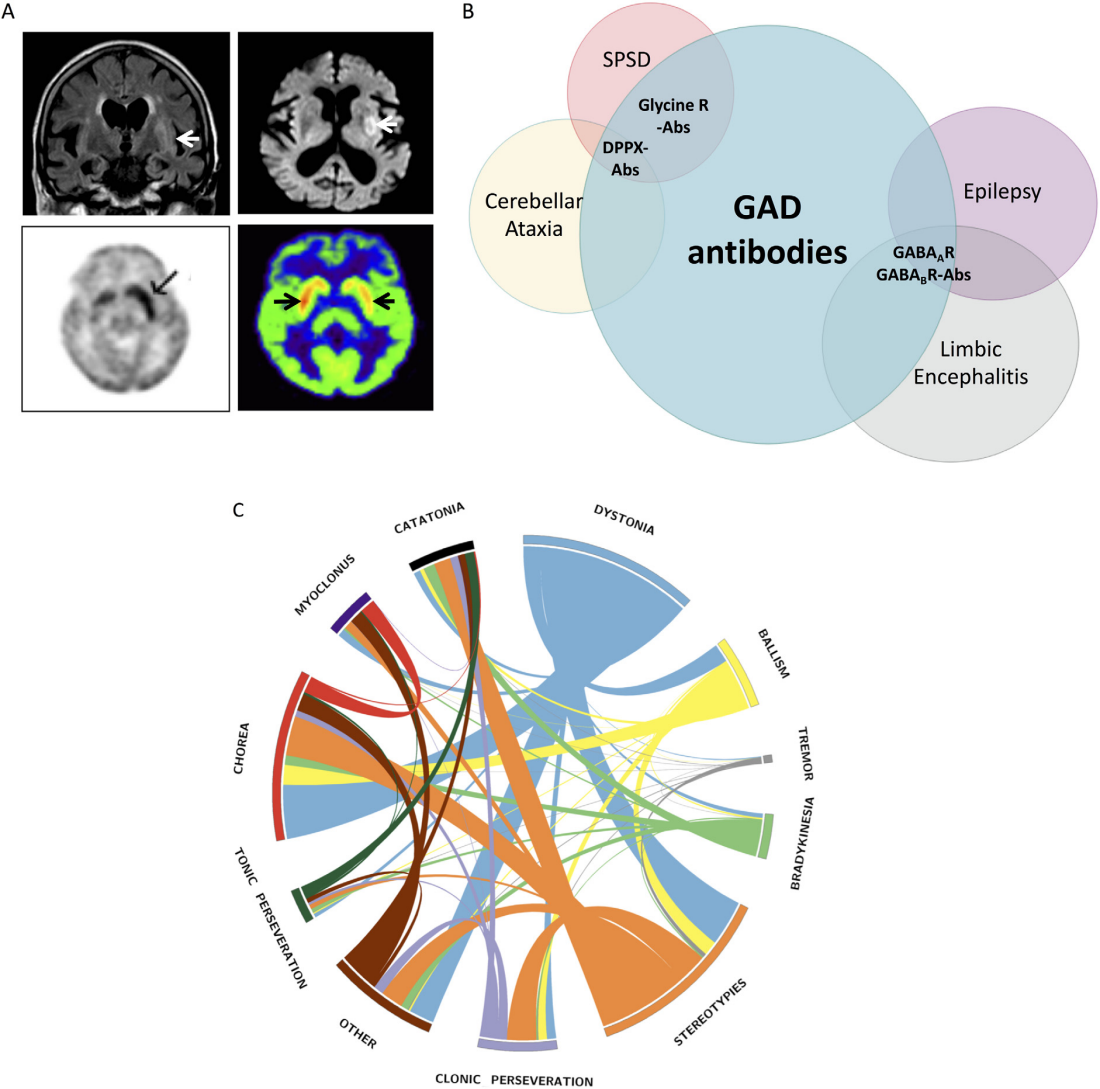
Radiological spectrum of faciobrachial dystonic seizures with LGI1‐antibodies and clinical spectrum of glutamic acid decarboxylase (GAD) antibodies. (A) Multimodal radiological involvement of the basal ganglia in patients with LGI1‐antibodies and faciobrachial dystonic seizures using FLAIR and DWI‐weighted MRI (top 2 panels), PET (bottom left panel), and SPECT (bottom right panel) imaging. Arrows indicate abnormal basal ganglia regions. Reproduced with permissions.^20^, ^49^, ^52^ (B) Spectrum of overlapping autoimmune neurological diseases associated with GAD65 antibodies and concommittant CNS‐specific autoimmunity. Autoantibodies highlighted in bold. DPPX, dipeptidyl‐peptidase‐like protein‐6; LGI1, leucine‐rich glioma‐inactivated‐1, FLAIR, Fluid Attenuated Inversion Recovery; DWI, diffusion‐weighted imaging; SPECT, single‐photon emission computed tomography. (C) Circos diagram depicting the relative presence of phenomenological features in patients with NMDAR‐antibody encephalitis (adapted from Varley et al.^29^)

The major factor that governs the likely pathogenicity of an autoantibody response is whether it targets the intracellular versus extracellular domain of the autoantigen (Table 1 and Fig. 1). Autoantibodies directed against the extracellular domains of surface proteins (NSAbs; often termed *neuroglial surface autoantibodies*) are able to exert an effect on their target antigen in vivo and are therefore considered to have pathogenic potential. In contrast, autoantibodies that target intracellular antigens may never have the opportunity to bind their target.^8^, ^9^, ^10^ Many such autoantibodies (including Hu, Yo, Ri, CRMP5, and Ma2) are considered bystanders of an immunological process that is often mediated by pathogenic CD8 (cluster of differentiation) T cells.^11^, ^12^ These autoantibodies are frequently associated with tumors and are summarized in Table 1 and previous reviews.^3^, ^13^ Indeed, unlike NSAb‐related disorders,^14^ the passive transfer of intracellular‐directed autoantibodies to experimental animals has failed to reproduce features of the disease.^15^ Furthermore, it may be predicted that if driven by a dominant CD8 T cell response, the human disease should benefit from drugs directed to inhibit T cell function and, indeed, sirolimus has been shown to somewhat improve functional outcomes in these disorders that typically have a very poor prognosis.^16^ In addition, a hinterland category exists of antigens that may transiently reach the cell surface, such as those directed at the synaptic vescicle protein glutamic acid decarboxylase (GAD, Fig. 3). Here, exposure to the extracellular compartment may occur during vescicle fusion and potentially account for an effect on the intracellular antigenic target.^8^ Alternatively, they may represent an immune epiphenomenon that is sometimes detectable alongside, currently largely unidentified, coexistent NSAbs.^17^ In addition, antibodies to the voltage‐gated potassium channel complex that do not target leucine‐rich glioma‐inactivated‐1 (LGI1) or contactin‐associated protein‐like 2 (CASPR2) are directed against intracelluar epitopes and, associate with a broad, diverse, and seemingly unrelated set of neurological conditions.^18^ Therefore, these clinically‐irrelevant ‘double‐negative’ voltage gated potassium channel complex antibodies will not be discussed further herein.

## Treatment Principles

Unlike most of those with solely intracellular‐directed autoantibodies, patients with NSAbs often show a good response to first‐line therapies such as corticosteroids, intravenous immunoglobulins, and plasma exchange.^19^, ^20^, ^21^ Patients with GAD antibodies can also show a response to many of these treatments, but overall this cohort is more refractory to available agents.

However, in all patients, the upscaling of immunotherapies to second‐line treatments—such as cyclophosphamide and rituximab—should be instigated in those refractory to the first‐line therapies. This up‐titration should be quicker in patients whose disease is more severe, often within 2 weeks in *N*‐methyl‐d‐aspartate receptor (NMDAR)‐antibody encephalitis. Also, there are increasingly strong data to support the generic notion that early treatments improve clinical outcomes^19^, ^21^, ^22^, ^23^, ^24^, ^25^; hence, therapies should be ideally instigated on the basis of a clinical diagnosis while awaiting confirmatory serology.

Therefore, in this review, we emphasize highly distinctive clinical features within the autoantibody‐associated movement disorders and use the aforementioned framework to reflect on the underlying mechanistic neuroimmunology as a basis to guide current and future immunotherapy options.

## The NSAb‐Mediated Syndromes and Their Related Immunology

### NMDAR Antibodies

#### *Clinical Features*

The discovery of autoantibodies against the GluN1 (NR1) subunit of the NMDAR identified a diffuse encephalitis with early psychiatric and cognitive features.^19^, ^24^, ^25^, ^26^ The associated characteristic hyperkinetic movement disorder is typically recognized after about 1 to 2 weeks, commonly involves the face, limbs, and trunk and has been variably described as dyskinetic orchoreoathetoid.^24^, ^25^, ^27^, ^28^ However, a recent study has suggested a more complex, combinatorial nomenclature may be most appropriate with expert raters noting the highly‐distinctive combination of dystonia, chorea and stereotypies with a paucity of tremor or myoclonus in many patients (Fig. 3C).^29^ Accompanying agitation may alternate with periods of catalepsy and catatonia and sometimes a hypokinetic phenotype can predominate, resembling endophenotypes of encephalitis lethargica.^30^

#### *Treatment*

In NMDAR‐antibody encephalitis, around 50% of patients respond to first‐line medications, usually with a good recovery over several weeks to months.^19^, ^24^ However, in this condition, their use may be limited by agitation and behavioral difficulties. This is especially true of plasma exchange, which requires significant patient compliance. Hence, it is sometimes necessary to sedate patients to permit administration of these first‐line therapies. For patients who are refractory to these drugs, rituximab and/or cyclophosphamide are recommended second‐line options, and there are data to suggest that their administration is associated with improved outcomes.^19^, ^22^, ^24^, ^25^ Throughout, early removal of the ovarian teratoma should be a therapeutic goal. As recent laboratory observations may explain some of these clinical findings,^31^, ^32^, ^33^ we next discuss the immunology in the context of the therapeutic data.

#### *Immunology*

NMDAR‐antibody encephalitis is associated with 2 known immunological triggers: an ovarian teratoma and preceding herpes simplex virus encephalitis (HSVE). Although in patients with intracellular directed autoantibodies tumors are often malignant, the ovarian teratoma in patients with NMDAR antibodies is typically benign. The teratoma, seen in about 20% of adults and few children,^24^, ^25^, ^30^ is likely to be a site of immunization as it contains dense infiltrations of T cells and B cells^31^ and its removal can hasten recovery.^19^, ^24^ Indeed, a recent paper showed that lymphocytes—both B cells and plasma cells—within the teratoma have the capacity to produce NR1‐directed autoantibodies, and the cystic teratoma fluid contains higher levels of NMDAR‐antibodies than serum.^31^

By contrast, the mechanism of NMDAR‐antibody encephalitis post‐HSVE is less clear.^34^, ^35^, ^36^ Typically, this disorder begins around 4 to 8 weeks after onset of HSVE at a time where patients, mainly children, are improving from the HSVE. Children often present with prominent choreoathetosis, abnormal behavior, and cognitive impairment: this syndrome appears identical to a primary NMDAR‐antibody encephalitis and distinctive from a relapse of HSVE.^37^ In adults, a similar pathophysiological phenomenon is observed but is not associated with a clear clinical relapse, rather a prolonged cognitive syndrome associated with a lower rate of abnormal movements.^38^, ^39^ Mechanistically, the necrotic disease process of HSVE may release a variety of neuronal antigens, including neuronal surface proteins. This may be more prominent after HSVE and other viruses, by comparison to traumatic brain injury, stroke, or neurodegeneration^40^ due to the more inflammatory environment or direct effects of viruses on lymphocytes.^41^ Subsequently, released antigen may be soluble or taken up by antigen‐presenting cells that migrate to cervical lymph nodes, the secondary lymphoid organs known to drain CNS lymphatics.^42^ Presentation of this antigen to T cells can lead to consequent B cell activation and antibody production in lymph node germinal centres (Figure 2). Consistent with this interaction, interruption of germinal center reactions with ongoing T cell and B cell interactions may explain the benefits of early and rapidly escalated immunotherapies including corticosteroids, cyclophosphamide, and rituximab.^19^, ^24^, ^25^, ^43^ Indeed, circulating B cells from patients with NMDAR antibody encephalitis can produce NMDAR antibodies in vitro, especially under conditions that mimic T cell help.^31^ Methods to determine the degree and nature of T and B cell involvement may in future help predict the value of cell specific therapies: for example, the autoantibodies can be transiently removed with plasma exchange, B cells deleted with rituximab, and the T cells inhibited with drugs such as cyclophosphamide.

In terms of autoantibody generation mechanisms, molecular mimicry between HSV‐associated antigens and the NMDAR seems unlikely as other CNS viruses, such as varicella zoster, have been shown to trigger NMDAR‐antibody encephalitis.^44^ By analogy, we have also observed NMDAR‐antibody encephalitis after other viral and idiopathic neurological inflammatory illnesses (Irani and Leite, 92). In addition, there is often the concomitant presence of other antigen‐specific NSAbs, such as those against the dopamine 2 receptor, gamma‐aminobutyric acid type A receptor (GABA_A_R) and other unknown targets, after HSVE.^35^, ^45^ Finally, no viral epitope has been reported with sequence homology to the NMDAR. Rather, this array of autoantibody specificities post‐HSVE is likely to reflect the concept of epitope spread, where, in an inflammatory milleu, there is a polyclonal immune response against a range of antigens exposed after a single inciting event. However, given that most patients have neither preceding HSVE nor a teratoma and this idiopathic group have the highest relapse rate,^19^, ^24^ the most common immunological triggers of this condition have clinical importance and await discovery. One such trigger may be genetic, perhaps a HLA predisposition, particularly given the known nonwhite racial bias of this condition.^24^, ^46^

By contrast to emerging data about the cellular immunology, the autoantibodies themselves have been relatively well characterized. Their principle mechanism of action appears to be the downregulation of surface NMDARs. This leads to a direct reduction infunctional NMDARs and, in addition, may have consequences for the stability and function of other neighboring synaptic and extrasynaptic proteins.^25^, ^47^ Furthermore, although the NMDAR antibodies are of the complement‐fixing IgG1 subclass, the available brain pathology does not show complement deposition.^24^, ^48^ Complement induction often causes tissue necrosis. Hence the absence of complement deposition, alongside established functional effects of the autoantibodies, may explain the substantial reversibility and limited atrophy observed in this condition after immunotherapy.^19^, ^24^, ^25^

### LGI1 Antibodies

#### *Clinical Features*

Faciobrachial dystonic seizures (FBDS) are stereotyped, frequent, and brief dystonic movements consistently associated with LGI1 antbodies (see Supporting Information Video). They predominantly involve the arm and the ipsilateral face, and less commonly the leg or the trunk.^20^, ^21^, ^49^ As the attacks are rarely associated with disturbance of consciousness or ictal EEG changes, they may be considered to lie in a borderland between movement disorders and seizures.^50^ However, and consistent with seizures, FBDS can be preceded by sensory auras and automatisms, and both agitation and speech arrest are described during or after the episodes.^20^, ^49^ Nevertheless, the semiology of FBDS is very different to that of more typical frontal and temporal lobe epilepsies. The attacks show a limited response to antiepileptic drugs, and multimodal radiological involvement of the basal ganglia is frequently observed in patients with FBDS (Fig. 3A).^20^, ^49^, ^51^, ^52^ Therefore, the origin of FBDS, and their preferred classification as a movement disorder or an epilepsy, is still debated, but pragmatically FBDS certainly continue to present to movement disorder neurologists, amongst others. Importantly, although FBDS were originally observed in the context of marked cognitive impairment (as part of an “encephalitis”), several patients present with FBDS alone. Furthermore, the relatively dramatic response of FBDS to immunotherapies, and their onset preceding the development of cognitive impairment, led to the hypothesis that their effective cessation may prevent the occurrence of cognitive impairment associated with limbic encephalitis.^20^, ^49^ Indeed, in a recent cohort of 103 patients with FBDS, this appeared to be the case, with cognitive impairment appearing frequently, and almost exclusively, in patients with ongoing FBDS.^21^ This may also be true of other seizure semiologies in patients with LGI1‐antibodies, which are well‐recognised and also very frequent.^53^, ^54^, ^55^ Moreover, as generalized chorea can sometimes precede the onset of LGI1‐antibody encephalitis, perhaps a similar paradigm also operates in this clinical scenario.^56^, ^57^

#### *Treatment*

Therefore, after a clinical diagnosis is made, and by analogy to NMDAR antibodies, early immunotherapy appears to be key to outcome optimization.^21^ Timing is especially critical in this condition as there has been a demonstrable reduction in the probability of seizure cessation with each day of delay to immunotherapy, and because the effective treatment of FBDS may prevent cognitive impairment. In this condition, there are surprisingly limited data to suggest a benefit of rituximab.^58^ However, to date, early treatment with rituximab has not been systematically reported, nor have studies using medications including cyclophosphamide or bortezomib.

#### *Immunology*

The highly consistent association between a distinctive clinical phenotype and the presence of LGI1 antibodies strongly suggests that they have a pathogenic role. This has been strengthened by in vitro data that implicate alpha‐amino‐3‐hydroxy‐5‐methyl‐4‐isoxazolepropionic acid (AMPA)‐receptors and potassium channels in the downstream mechanisms of LGI1‐modulation induced neuronal dysfunction.^10^, ^59^, ^60^ Other functional effects of the LGI1 autoantibodies include the downregulation of the LGI1 complex, which includes LGI1's natural binding partners a disintegrin and metalloproteinase domain‐containing protein 22 and 23 (ADAM22/23).^21^ In addition, and by contrast to the NMDAR‐directed antibodies, LGI1 antibodies are mainly of the IgG4 subclass.^21^, ^61^ However, the LGI1‐IgG1 antibodies appear to correlate with disease severity, perhaps as they have the potential to deposit complement in the brain, as observed from some postmortem tissues.^21^, ^62^ Although tumors and prodromal infections have not been consistently observed in patients with LGI1 antibodies, the recent description of an almost universal HLA‐DRB1*07:01 allele strongly implicates a role for T cells in disease pathogenesis.^63^, ^64^ These LGI1‐specific T cells are likely to interact with B cells in peripheral germinal centers.

### CASPR2 Antibodies

#### *Clinical Features*

Antibodies against the juxtaparanodal protein CASPR2 are associated with a variety of movement disorders including neuromyotonia, chorea, ataxia, and a syndrome of orthostatic myoclonus.^3^, ^65^ Many of these typically occur in the context of an encephalopathy that is similar to that associated with LGI1 antibodies. Indeed, although neuromyotonia is often thought to occur as an isolated phenomenon, it is intriguing that many patients have additional autonomic and CNS features, suggesting a frequently more diffuse neuronal disease process.^66^

#### *Treatment*

The management of CASPR2‐antibody conditions has received little attention to date.^67^, ^68^, ^69^ Our experience suggests that the ataxia and encephalopathy are usually responsive to similar therapeutic approaches as for patients with LGI1‐antibody encephalitis (Irani, Jacob, & Leite, 2017).

#### *Immunology*

As with LGI1 antibodies, one plausible mechanism of CASPR2‐antibody‐induced hyperexcitability is interference with the tightly CASPR2‐complexed juxtaparanodal potassium channels.^10^ This mechanism has been suggested by human models of CASPR2 mutations and was recently confirmed in animals receiving CASPR2‐IgG.^70^ In terms of autoantibody generation, CASPR2 antibodies are often associated with a thymoma, particularly in patients with neuromyotonia and Morvan's syndrome,^61^ and this frequent clinical observation may implicate defective central tolerance checkpoints that permit autoreactive T cells to escape into the periphery and facilitate antigen‐specific autoimmunity. In addition, 50% of patients with CASPR2‐antibodies have a recently described HLA (human leucocyte antigen)‐DRB1*11:01 association, which contrasts to the HLA‐DRB1*07:01 allele observed in patients with LGI1‐antibodies.^64^

### Aquaporin‐4 (AQP4) and Myelin Oligodendrocyte Glycoprotein (MOG) Antibodies

#### *Clinical Features*

Another distinctive paroxysmal phenomenon is the tonic spasms observed in neuromyelitis optica spectrum disorders (NMOSD). Tonic spasms occur more frequently in NMOSD than multiple sclerosis^71^ and consist of recurrent, painful, asymmetrical dystonic posturing, typically in one or more limbs, that usually last a few seconds to minutes and occur at high frequency.^72^ Occasionally, they can be preceded by a sensory aura and frequently they are triggered by hyperventilation, tactile stimuli, or voluntary movements. Typically, patients have a favorable course with a rapid response to anticonvulsant drugs.

Patients with the more recently described MOG antibodies typically associate with phenotypes of optic neuritis, longitudinally extensive myelitis, and acute disseminated encephalomyelitis.^73^ Although the latter often shows basal ganglia and thalamic imaging changes, there are only rare descriptions of movement disorders in patients with MOG antibodies in addition to the few with ataxia.^74^

#### *Treatment*

AQP4‐antibody‐mediated NMOSD is a chronic, naturally relapsing condition. Although immunotherapy efficacy has not been explored alongside a placebo arm, to date it appears that rituximab, azathioprine, and mycophenolate mofetil all reduce relapse rates by around 60% to 70%.^75^, ^76^ Furthermore, the avoidance of several agents with proven efficacy in multiple sclerosis is important in NMOSD as they can promote NMOSD relapses.^77^ The longer term treatment of MOG‐antibody‐mediated diseases has only recently been investigated, and it was revealed that a corticosteroid duration of less than 6 months is associated with a higher rate of relapses.^73^

#### *Immunology*

The astrocytopathy associated with NMOSD is characterized by autoantibodies directed against AQP4, a water channel expressed on the astrocyte end‐foot processes. These autoantibodies can induce complement deposition, AQP4 internalization, and cointernalization of the glutamate transporter excitatory amino acid transporter‐2 (EAAT2).^78^ The deposition of complement is marked in tissue from patients with NMOSD and is a likely explanation as to why the relapses can produce severe disability. Indeed, a recent study using the monoclonal antibody eculizumab, which neutralizes the C5 complement component, has shown striking efficacy.^79^ By contrast to AQP4 antibodies, the initial pathology induced by MOG antibodies occurs on oligodendrocytes, and the downstream mechanisms are currently under active investigation.

### IgLON5‐Antibody Associated Neurodegeneration

#### *Clinical Features*

Patients with Iglon5‐autoantibodies mostly present with a chronic history of a rapid eye movement sleep behavior disorder with a distinctive non–rapid eye movement parasomnia plus bulbar involvement, dysautonomia, stridor, and hypoventilation.^4^ The main movement disorder described is chorea, but a few cases with postural instability and a supranuclear vertical gaze palsy had a phenotype resembling progressive supranucelar palsy (PSP), and the clinical spectrum continues to expand with the recent inclusion of myoclonus, myorhythmia, and dystonia.^80^, ^81^

#### *Immunology*

IgLON family member 5 (IgLON5) antibodies are found in the serum and CSF of patients with postmortem evidence of a tauopathy. This finding highlights a novel relationship between autoimmune and degenerative disorders.^81^ IgLON5 antibodies bind the extracellular domain of their target neuronal protein and avidly label live neurons in culture. Furthermore, these patients have a consistent HLA‐DQB1*0501 and HLA‐DRB1*1001 genotype association. However, the phenotype and histology are highly suggestive of a neurodegenerative process. Brain pathology shows neuronal loss and extensive deposits of hyperphosphorylated tau protein predominantly in the hypothalamus and the brain stem tegmentum, with a different distribution from other tauopathies.

#### *Treatment*

Although initial reports described a universally poor outcome after immunotherapy, with death a common outcome,^81^, ^82^ more recent work reports a relatively frequent response to immunotherapies.^80^ It may be that earlier recognition of this condition portends a promising outcome with immunotherapies. In summary, this distinctive tauopathy is associated with NSAbs and a clear HLA association and may be a paradigm for the future study of the immune system leading to neurodegeneration. If so, it may yet be that all known NSAbs are pathogenic.

Indeed, in the other NSAb‐associated diseases discussed previously, the autoantibodies are also very likely to be causative. Hence, knowledge of the location and subsets of B cells that produce these autoantibodies has biological and therapeutic relevance.^31^, ^83^ Therefore, next we use conventional immunological paradigms to help describe and model mechanisms of antigen‐specific autoantibody generation in these conditions and link these to current and future immunotherapies.

## The Therapeutically Relevant Immunology

Identifying the autoantibody‐producing cells has important potential implications for considering future individualized therapies because, as shown in Figure 2, through the B cell lineage there are different sets of expressed surface markers. These markers alter as originally naïve B cells encounter T cell help and antigen, and differentiate into class‐switched IgG‐positive memory B cells.^84^, ^85^ Spatial requirements for these interactions are met in germinal centers, and this interaction involves a number of T and B cell molecules—both inhibitory and stimulatory—that regulate the intensity of this reaction.^86^ In humans, this balance is well exemplified by the appearance of autoantibody‐mediated neurology after administration of T cell–directed checkpoint inhibitors.^87^, ^88^ Therefore, even in the most well‐established NSAb‐mediated diseases, an isolated contribution of B cells is unlikely, and there should be more consideration given to therapies targeting both T cells and B cells.

Successfully activated B cells will typically undergo successive rounds of interactions with antigen and T cells until they acquire high‐affinity antibodies. Subsequently, these activated B cells may differentiate into antibody secreting cells in circulation (plasmablasts), where importantly they downregulate CD20, the target of rituximab.^89^ The plasmablasts which reach bone marrow niches have also often downregulated CD19 and expressed CD138.^90^ One important question is the degree to which these now long‐lived bone marrow resident plasma cells produce the autoantibodies.^85^ If they are major producers of autoantibodies, patients should be sensitive to drugs such as bortezomib, which target the proteasome—an organelle that is highly active in plasma cells. Indeed, early observational studies suggest a possible, albeit limited, response to bortezomib.^91^ Conversely, rituximab should have little impact as these cells are CD20 negative. Yet there is evidence that several such syndromes can respond to CD20‐targeted medications.^19^, ^58^, ^76^ Therefore, further careful clinic‐immunological studies are required to highlight the relative roles of these therapies and their effect on B cell subsets and autoantibody levels: these will better inform our understanding of disease immunobiology.^31^, ^32^, ^33^, ^83^ It may be that combinations of plasma cell depleting agents plus removal of precursor B cells are required to effectively remove autoreactive capacities from the B cell lineage.

However, it is not solely the lymphocyte surface markers that provide therapeutically tractable drug targets. There are increasing examples where pharmacological manipulation of the cytokine and chemokine signalling pathways produce therapeutic efficacy. For example, B cells and antibody‐secreting cells are known to show dependence on Interleukin 6 (IL‐6) for survival. Indeed, inhibition of the IL‐6 receptor with tociluzimab has some proven benefit in both NMOSD and NMDAR‐antibody encephalitis.^93^, ^94^ Perhaps this line of inquiry will generate further targets to which there are already available modulators.

As causative autoantibodies are consistently, albeit not universally, found in the CSF, one possibility is disease initiation and propagation by a primary intrathecal response: a hypothesis that does not involve peripheral lymph nodes. This is strongly mitigated by the consistent observation of serum autoantibodies in all of these conditions, including NMDAR‐antibody encephalitis post‐HSVE, typically at concentrations far in excess of the CSF autoantibodies.^1^, ^10^, ^24^, ^34^, ^95^, ^96^ In addition, the universal observation of peripheral, likely immunizing, systemic tumors also mitigates this possibility. More plausible is the notion of an initiating peripheral immunization. Perhaps in a manner akin to HSVE, many CNS‐restricted antigens reach the periphery via CNS lymphatics and draining cervical lymph nodes (Fig. 2).^42^ The constitutive versus active nature of this drainage requires further investigation as it may determine the probability of a CNS lesion initiating a peripheral immunization.

Subsequent to the immunization, at least some of the peripheral response must transfer to the CNS to mediate a brain disease. One outstanding question is whether this is principally mediated by migration of the B cells or the soluble antibodies across the blood‐brain or blood‐CSF barrier. This distinction may alter clinical management strategies; for example, natalizumab will block lymphocyte trafficking across the blood‐brain barrier. Several clues exist to help us understand the nature of the brain‐based immune response. Animal studies lend support to the intuitive notion that the brain‐expressed antigens can act as a “sink” for CNS‐transferred serum autoantibodies resulting in undetectable CSF autoantibody levels.^97^ Indeed, although CSF LGI1 antibodies are present in most patients, they are undetectable in some cases,^96^, ^98^ and this balance may represent a saturation point of the “sink.” By contrast, in patients with NMDAR‐antibody encephalitis, the presence of autoantibodies in CSF is a requirement for definitive diagnosis.^99^ However, serial measurements of CSF or serum autoantibody levels in all of these conditions only broadly correlate with clinical outcomes,^19^, ^20^, ^100^ and perhaps other, many as yet undetermined, factors including cytokines, complement and even other autoantibody reactivities,^33^ together contribute to the overall clinical status. In addition to diffusion alone, there is often marked intrathecal synthesis of the NMDAR antibodies and, indeed, NMDAR‐autoantibody secreting cells within the CNS have recently been isolated by single‐cell cloning techniques.^33^ Therefore, the intrathecal retention of antigen‐specific cells appears necessary for generation of NMDAR‐antibody encephalitis and may be mediated by the high CSF levels of the B cell and plasma cell chemokine (C‐X‐C motif) ligand 13 (CXCL13).^32^ These observations, coupled with the highly heterogeneous phenotypes of patients with NMDAR antibodies in serum but not CSF,^40^, ^101^, ^102^ may suggest that the predicted natural diffusion of NMDAR‐IgG into the CNS is insufficient to generate this encephalitis phenotype. Yet, maybe the soluble autoantibodies do play a role in ongoing disease as plasma immunoabsorption of IgG leads to a fall in CSF autoantibodies and correlates with improvements in clinical status.^103^ The relative contributions of serum autoantibody transfer into the CNS and the degree of immune cell infiltration may vary across diseases but also within diseases, and could determine the likelihood of amelioration with plasma exchange or predict the need for future intrathecal‐directed therapies.

### Autoantibodies Against Glutamic Acid Decarboxylase 65 (GAD65), an Intracellular Synaptic Protein: Syndromes, Immunology, and Treatments

In contrast to the NSAb‐mediated disorders, those associated with autoantibodies directed against intracellular targets are generally considered nonpathogenic. Indeed, as discussed previously, passive transfer and active immunization experiments have proven that some of these autoantibodies do not cause neurological diseases.^104^ However, the spectrum of disorders associated with autoantibodies against GAD65 and amphiphysin may challenge this notion from clinical and laboratory perspectives.^105^, ^106^ Here, we discuss the GAD‐antibody syndromes, with prominent movement disorders, in greater detail.

#### *Clinical Features*

Antibodies against the intracellular enzyme GAD65 are very frequently detected in stiff person syndrome and related disorders (stiff person spectrum disorders [SPSD]). This group of disorders share the hallmark features of fluctuating muscle stiffness with superimposed spasms and hyperekplexia (an excessive startle response to acoustic or tactile stimuli).^107^ Classic stiff person syndrome involves stiffness of the lower back and proximal leg muscles with characteristic hyperlordotic posturing. In focal forms of SPSD, stiffness may be restricted to 1 limb (stiff limb syndrome).^108^ Other variants of SPSD are defined by the presence of additional neurological signs such as cerebellar ataxia. Progressive encephalomyelitis with rigidity and myoclonus typically designates the severe end of the spectrum, characterized by prominent hyperekplexia and myoclonus, generalized stiffness, brain stem signs, and dysautonomia.^109^

#### *Immunology Including Coexistent NSAbs*

High concentrations of GAD65 antibodies associate with a limited set of clinically distinctive phenotypes, namely: SPSD, cerebellar ataxia, epilepsy and limbic encephalitis (Fig. 3B), suggesting some syndrome specificity.^107^, ^110^, ^111^ In addition, several patients with GAD65 antibodies do respond to immunotherapy. Also, by comparison to patients with Hu‐ or Ma2‐antibody‐associated encephalitis, patients with GAD65‐antibody encephalitis showed lower CD8/CD3 ratios, indicating an appropriate designation of GAD65 antibodies between other intracellular autoantibodies and NSAbs.^62^ These collective observations lead to the intriguing notion that GAD65 antibodies may have some causative potential. Indeed, the closely related amphiphysin antibodies, typically associated with paraneoplastic stiff person syndrome, have been shown to both reproduce disease upon transfer to experimental animals, and they may gain access to their intracellular antigenic target.^8^, ^106^, ^112^

Alternatively, maybe the GAD65 antibodies coexist with NSAbs that target the extracellular domains of antigens at GABAergic and glycinergic inhibitory synapses, such as the alpha 1 subunit of the glycine receptor (GlyRα1), and GABA_A_R.^3^, ^105^, ^106^, ^107^ Furthermore, another NSAb found in some SPSD patients is directedagainst dipeptidyl‐peptidase‐like protein‐6 (DPPX),^113^, ^114^ a regulatory subunit of Kv4.2 potassium channels. Perhaps these coexistent autoantibodies, often with antigens expressed in the same neurons, implicate epitope spread as a mechanism to diversify the polyclonal immune responses after a triggering event that exposes several antigens to the immune system. Indeed, it appears that these coexistent NSAbs confer even more disease specificity: for example, Glycine Receptor (GlyR) antibodies frequently associate with progressive encephalomyelitis with rigidity and myoclonus and DPPX antibodies with a distinct phenotype of truncal stiffness, prominent hyperekplexia, and cerebellar ataxia.^113^ Both GlyR and DPPX are surface expressed, and the respective NSAbs are likely to be pathogenic, with in vitro evidence they induce antigen internalization.^109^, ^114^

#### *Treatment*

Consistent with the aforementioned paradigm of intracellular antibodies versus NSAbs, from patients with SPSD the treatment responses appear better in patients with GlyR antibodies than GAD65 antibodies alone.^115^ The treatment with the only proven randomized clinical data within the conditions discussed in this review is intravenous immunoglobulins in SPSD.^116^ Patients often show a moderate benefit from this drug, but longer term alternatives are yet to be satisfactorily explored.

## Future Directions

Given that our understanding of the immunology underlying NSAb‐mediated diseases remains in its infancy, this appears to be an important avenue for future study. Available data have led researchers to consider drugs that appear biologically intuitive, but it is yet to be seen if we can achieve disease specificity. There are some potential reasons to maintain optimism in the potential for precision medicine. First, the generation of patient‐derived monoclonal antibodies in some of these conditions offers a method to directly out‐compete the endogenous patient antibodies.^117^ Of course, this comes with a series of potential immunological hazards, but it would form an elegant method to test the hypothesis of whether the antibodies are the major disease perpetrators. Other options in the pipeline include the use of selective cytokine and chemokine blockade. For example, in NMDAR‐antibody encephalitis, raised CSF levels of CXCL13 have been proposed as an intrathecal lymphocyte chemoattractant. Their neutralization may inhibit lymphocyte crossing. However, if this were effective, it may yet require a parallel peripheral depletion of B cells to adequately extinguish the disease. Indeed, this combinatorial approach may be a future theme in these increasingly complex diseases that require collaborations of at least T cells and B cells. One such vision may by combinatorial assessment of the autoantibody production from patient lymphocytes under a large number of cytokine conditions^31^, ^83^ and then block the dominant culprit pathways with targeted monoclonal therapies. Such an approach would be patient specific, especially if complemented by evaluation of endogenous cytokine levels, but if generic stimuli expanded these B cells, this approach may be intrinsically limited. Given recent advances in the clinical immunology,^31^, ^83^, ^93^ future studies should be able to answer these possibilities rapidly.

## Conclusions

The increasing numbers of identified neuronal autoantibodies are associated with a broadening clinical spectrum of autoantibody‐mediated movement disorders. This contemporary expansion makes the field increasingly important for the movement disorder specialist and for the general neurologist. This is particularly the case given the recognition that early immunotherapy is likely to improve prognosis and prevent the ongoing pathogenic effects of the autoantibodies.

However, to understand the root causes of these illnesses, the field requires an improved future understanding of the varied roles of immune components—including T cells, B cells, plasma cells—and their associated surface markers. Indeed, many of the conventional immunological paradigms as outlined require confirmation with the direct study of these diseases. In addition to biological insights, this may offer a method to specifically target causative cell types in these diseases. The relative roles of these different immune components may vary in conditions with intracellular versus surface autoantibodies and depend on the inciting factor in the autoantibody‐mediated conditions. Furthermore, the degree to which reduction in T cell function or autoantibody level is required to achieve clinical improvement should be considered as many of these autoantibodies can persist for years despite good clinical remission.^20^, ^24^, ^118^ Therefore, to move toward precision medicine in these conditions, there is an urgency to better appreciate the immunological mechanisms that underlie the generation and perpetuation of the autoantibodies, and this may lead to novel therapeutic strategies that could be addressed in clinical trials.

## Author Roles

1) Research project: A. Conception, B. Organization, C. Execution; 2) Statistical Analysis: A. Design, B. Execution, C. Review and Critique; 3) Manuscript: A. Writing of the first draft, B. Review and Critique.

V.D.: 1A, 1B, 1C, 3A, 3B

B.B.: 1A, 1B, 1C, 3A, 3B

A.K.K.: 1B, 1C, 3B

S.R.I.: 1A, 1B, 1C, 3A, 3B

## Full financial disclosure for the previous 12 months

SRI is a coapplicant and receives royalties on patent application WO/2010/046716 (U.K. patent no., PCT/GB2009/051441) entitled ‘Neurological Autoimmune Disorders’. The patent has been licensed to Euroimmun AG for the development of assays for LGI1 and other VGKC‐complex antibodies.

## Supporting information

Supplementary Information
